# Targeting cancer-associated fibroblasts/tumor cells cross-talk inhibits intrahepatic cholangiocarcinoma progression via cell-cycle arrest

**DOI:** 10.1186/s13046-024-03210-9

**Published:** 2024-10-17

**Authors:** Serena Mancarella, Isabella Gigante, Elena Pizzuto, Grazia Serino, Alberta Terzi, Francesco Dituri, Eugenio Maiorano, Leonardo Vincenti, Mario De Bellis, Francesco Ardito, Diego F. Calvisi, Gianluigi Giannelli

**Affiliations:** 1 National Institute of Gastroenterology, IRCCS “S. de Bellis” Research Hospital, Via Turi 27, Castellana Grotte, BA 70013 Italy; 2 Division of General and Hepatobiliary Surgery, Department of Surgery, Dentistry, Gynecology and Pediatrics, University of Verona, G.B. Rossi University Hospital, P.le L.A. Scuro 10, Verona, 37134 Italy; 3 Hepatobiliary Surgery Unit, Foundation “Policlinico Universitario A. Gemelli”, IRCCS, Catholic University, Rome, Italy; 4 Institute of Pathology, University of Regensburg, 93053 Regensburg, Germany

**Keywords:** Cholangiocarcinoma progression, Tumor microenvironment, Gamma secretase inhibitor, Tumor stroma, Liver cancer

## Abstract

**Background:**

Cancer-associated fibroblasts (CAFs), mainly responsible for the desmoplastic reaction hallmark of intrahepatic Cholangiocarcinoma (iCCA), likely have a role in tumor aggressiveness and resistance to therapy, although the molecular mechanisms involved are unknown. Aim of the study is to investigate how targeting hCAF/iCCA cross-talk with a Notch1 inhibitor, namely Crenigacestat, may affect cancer progression.

**Methods:**

We used different in vitro models in 2D and established new 3D hetero-spheroids with iCCA cells and human (h)CAFs. The results were confirmed in a xenograft model, and explanted tumoral tissues underwent transcriptomic and bioinformatic analysis.

**Results:**

hCAFs/iCCA cross-talk sustains increased migration of both KKU-M213 and KKU-M156 cells, while Crenigacestat significantly inhibits only the cross-talk stimulated migration. Hetero-spheroids grew larger than homo-spheroids, formed by only iCCA cells. Crenigacestat significantly reduced the invasion and growth of hetero- but not of homo-spheroids. In xenograft models, hCAFs/KKU-M213 tumors grew significantly larger than KKU-M213 tumors, but were significantly reduced in volume by Crenigacestat treatment, which also significantly decreased the fibrotic reaction. Ingenuity pathway analysis revealed that genes of hCAFs/KKU-M213 but not of KKU-M213 tumors increased tumor lesions, and that Crenigacestat treatment inhibited the modulated canonical pathways. Cell cycle checkpoints were the most notably modulated pathway and Crenigacestat reduced CCNE2 gene expression, consequently inducing cell cycle arrest. In hetero-spheroids, the number of cells increased in the G2/M cell cycle phase, while Crenigacestat significantly decreased cell numbers in the G2/M phase in hetero but not in homo-spheroids.

**Conclusions:**

The hCAFs/iCCA cross-talk is a new target for reducing cancer progression with drugs such as Crenigacestat.

**Graphical abstract:**

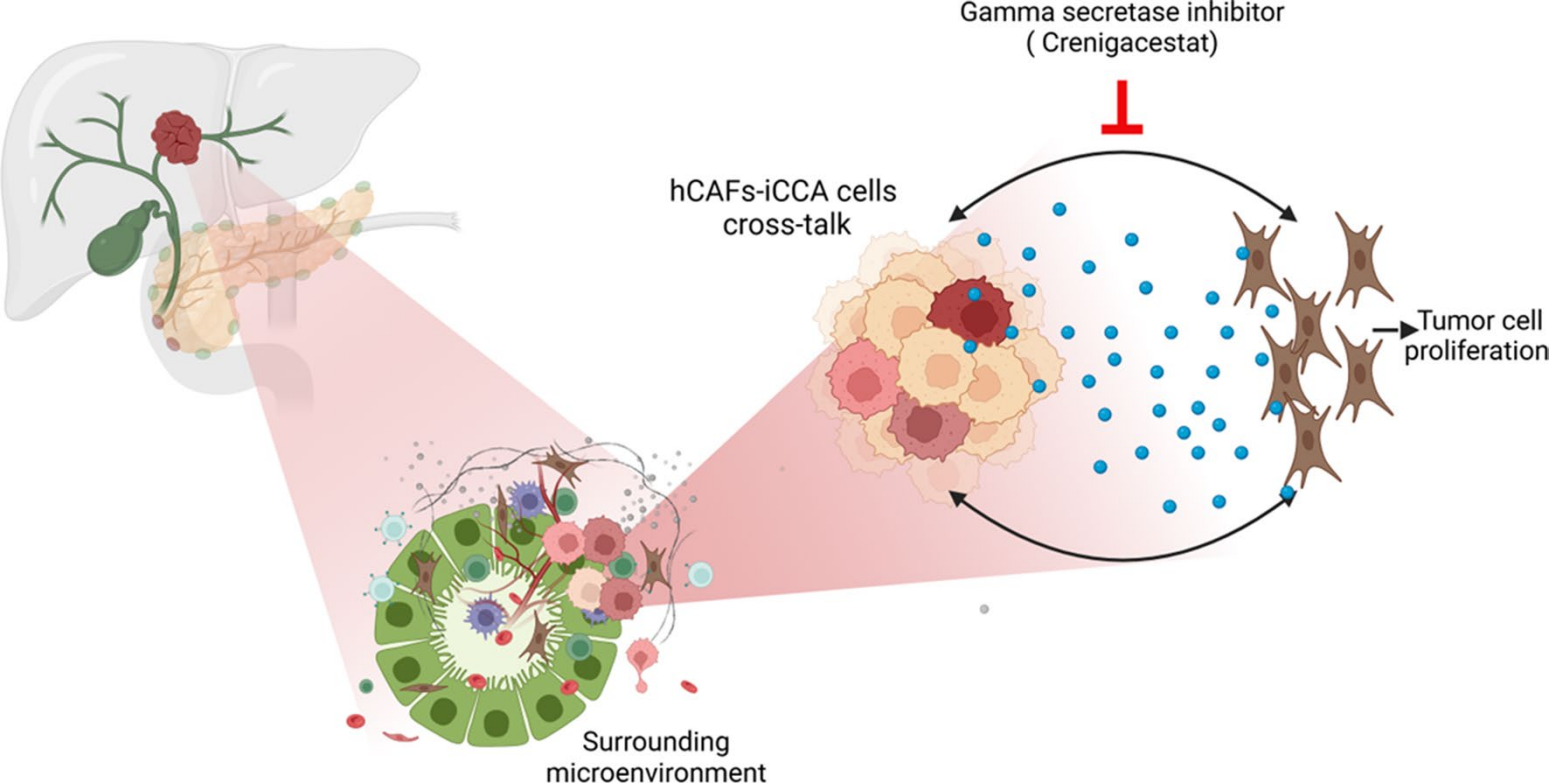

**Supplementary Information:**

The online version contains supplementary material available at 10.1186/s13046-024-03210-9.

## Introduction

In the last forty years, the incidence of intrahepatic Cholangiocarcinoma (iCCA) in the U.S. has increased by 128% [1]. Nevertheless, diagnostic skills, therapies and clinical management, responsible for prognosis and survival, have not significantly improved, and only approximately 20–30% of patients with iCCA are eligible for surgery [2], while most cases relapse, and best overall 5-year survival is not superior to 20% [3]. This makes iCCA a highly deadly disease, and, the lack of a reliable drug-based therapy for patients at an advanced stage of disease is a severe drawback for patients and clinicians. For these patients, gold standard therapy is still based on Gemcitabine-Cisplatin, Gemcitabine-Oxaliplatin (GEMOX) and 5-Fluorouracil (5-FU) [4–6]. However, chemotherapy is often only palliative, and this has driven the investigation of different targeted biological therapies. For instance, fusions at the Fibroblast Growth Factor Receptor gene (FGFR2) are reported in 10–15% of iCCA patients, for whom selective FGFR Tyrosine Kinase Inhibitors (TKIs) have been successfully tested, emphasizing once more the concept of precision medicine in iCCA [7, 8]. Overall, very little is known regarding the molecular mechanisms underlying iCCA progression, aggressiveness, and resistance to common chemotherapies.

The iCCA hallmark is the desmoplastic reaction, characterized by an abundant extracellular matrix (ECM) deposition, creating tissue boundaries that provide structural and biochemical support and leading to drug resistance [9–12]. Human Cancer Associated Fibroblasts (hCAFs) are a heterogeneous population of fibroblasts originating from various sources of resident fibroblasts, including stellate cells, that are mainly activated by Transforming Growth Factor (TGF)-𝛃1 and Platelet Derived Growth Factor (PDGF) that undergo the epithelial-to-mesenchymal transition (EMT), taking on a myofibroblast phenotype characterized by α-Smooth Muscle Actin (α-SMA) and Fibroblast Activation Protein (FAP) expression [13, 14]. hCAFs are mainly responsible for the ECM deposition and for remodeling the surrounding tissue microenvironment [15].

hCAFs establish a dynamic and mutual cross-talk with epithelial cancer cells, and are believed to contribute to tumor progression in different malignancies including iCCA [16–18]. To date, cytokines, growth factors, miRNA, freely released or carried by microvesicles are likely involved, but the mechanisms triggered by such a cross-talk are still unclear.

Notch pathway activation is triggered by direct cell-to-cell communication between Jagged (JAG 1, 2) or Delta- like (DLL 1, 3, and 4) ligands and Notch receptors (1–4), followed by cleavage of the Notch transmembrane domain by ɣ-secretase that leads to regulation of the transcription of target genes involved in cell proliferation, differentiation, and cell death. Aberrant Notch cleavage by ɣ-secretase has recently been demonstrated to be a main driver in the initiation and progression of iCCA [19]. Recently, we reported that Crenigacestat, a gamma secretase inhibitor, targets the NOTCH1/DLL4/VEGFA/MMP13 axis, reducing iCCA progression in a patient-derived xenograft (PDX) model by blocking tumor neovascularization [20]. We also demonstrated that Crenigacestat inhibits iCCA tumor progression in NOTCH1/HES1/THY1-positive xenograft models [21]. Moreover, in the surrounding ecosystem of iCCA, Crenigacestat inhibits the occurrence of liver fibrotic reactions by deactivating and reverting the myofibroblastic phenotype of iCCA hCAFs to a fibroblastic phenotype with a reduced secretion of TGF-β and ECM components such as FN, COL1A1, and COL1A2 [22].

The aim of this study is to investigate whether the hCAFs/iCCA crosstalk may be a suitable target for drugs such as Crenigacestat, and whether this could affect iCCA cancer progression.

## Results

### Effects of CAFs/iCCA cells cross-talk on migration and viability of iCCA cells

**Fig. 1:**
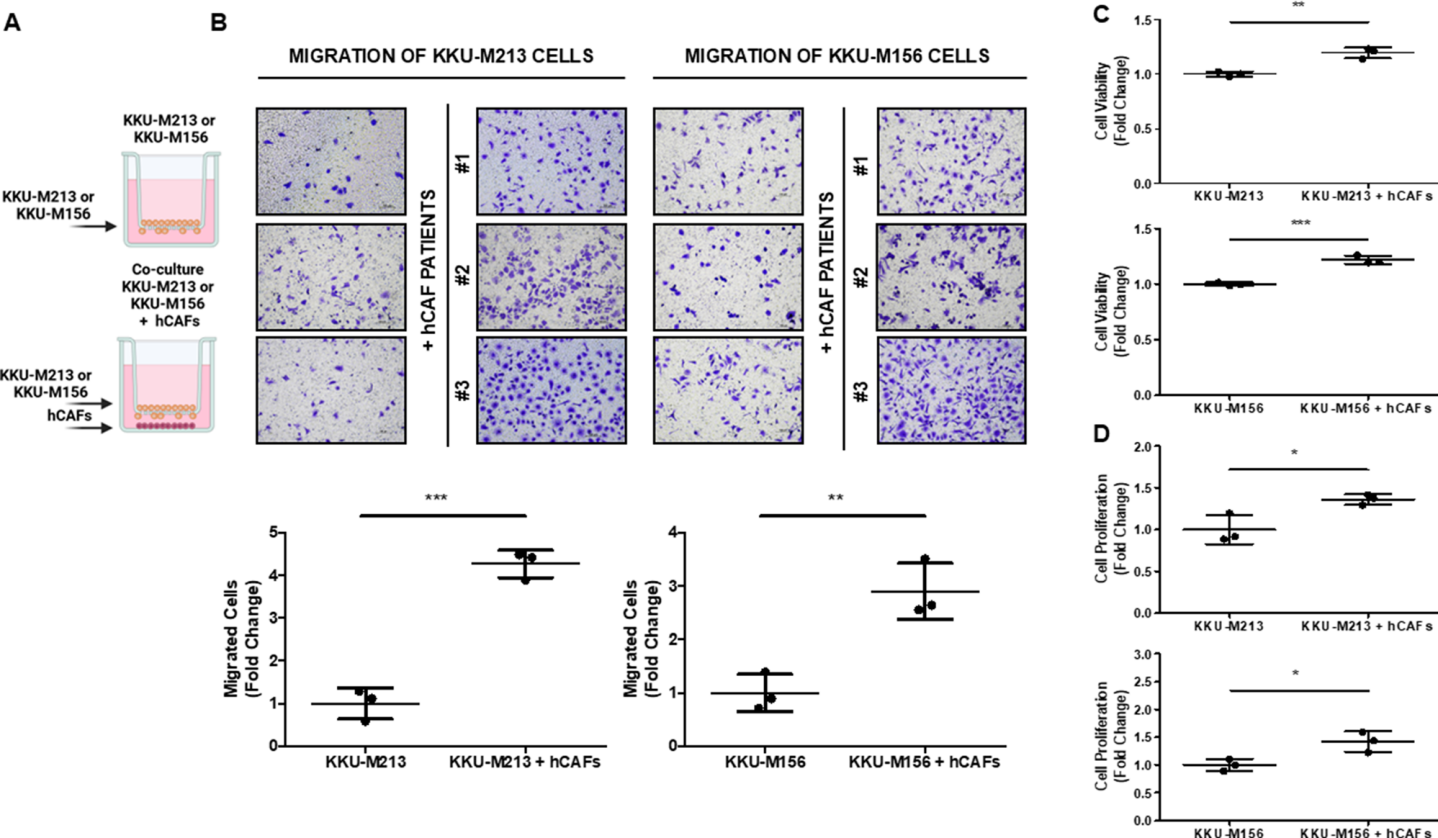
hCAFs promoted the migration and viability of iCCA cells in co-culture conditions. (**A**). Schematic representation of the experimental model of KKU-M213 and KKU-M156 migration without and with hCAFs. (**B**). Representative images of KKU-M213 (panels on the left) and KKU-M156 cells (panels on the right) migrated alone or in co-culture with three hCAFs (#1, #2, #3) using the transwell migration system. The migration of both iCCA cell lines was significantly increased after co-culture with hCAFs. Cells that migrated were counted in five random microscope fields for each sample (10× magnification, scale bar 100 μm). (**C**). The KKU-M213 and KKU-M156 cell viability was evaluated by CyQUANT™ XTT Cell Viability kit. The viability of both iCCA cells was significantly increased in co-culture with hCAFs. (**D**). The KKU-M213 and KKU-M156 cell proliferation was evaluated by Trypan Blue cell counting. Numbers of both iCCA cells were significantly increased in co-culture with hCAFs. Data are expressed as the mean ± SD of three independent experiments with three hCAFs. *p < 0.05 **p < 0.01 and ***p < 0.001

To investigate the role of the CAF/iCCA cells cross-talk, we challenged KKU-M213 and KKU-M156 iCCA cell lines to migrate in the presence of human hCAFs, isolated from three different patients who underwent surgery for iCCA. As reported in Fig. 1A-B, both KKU-M213 and KKU-M156 cell lines migrated on Coll I, more efficiently (*p* < 0.001 and *p* < 0.01) in the presence than absence of hCAFs seeded at the bottom part of the transwell. Consistently, the cell viability viability and proliferation of both KKU-M213 and KKU-M156, Fig. 1C-DB, was significantly (*p* < 0.01 and *p* < 0.001 for viability; and *p* < 0.05 for proliferation) increased in the presence compared to the absence of hCAFs. In conclusion, the hCAFs/iCCA cells cross-talk promoted the migration, viability and proliferation of iCCA cells.

### Crenigacestat effectiveness on migration induced by the hCAFS/iCCA cells cross-talk

**Fig. 2:**
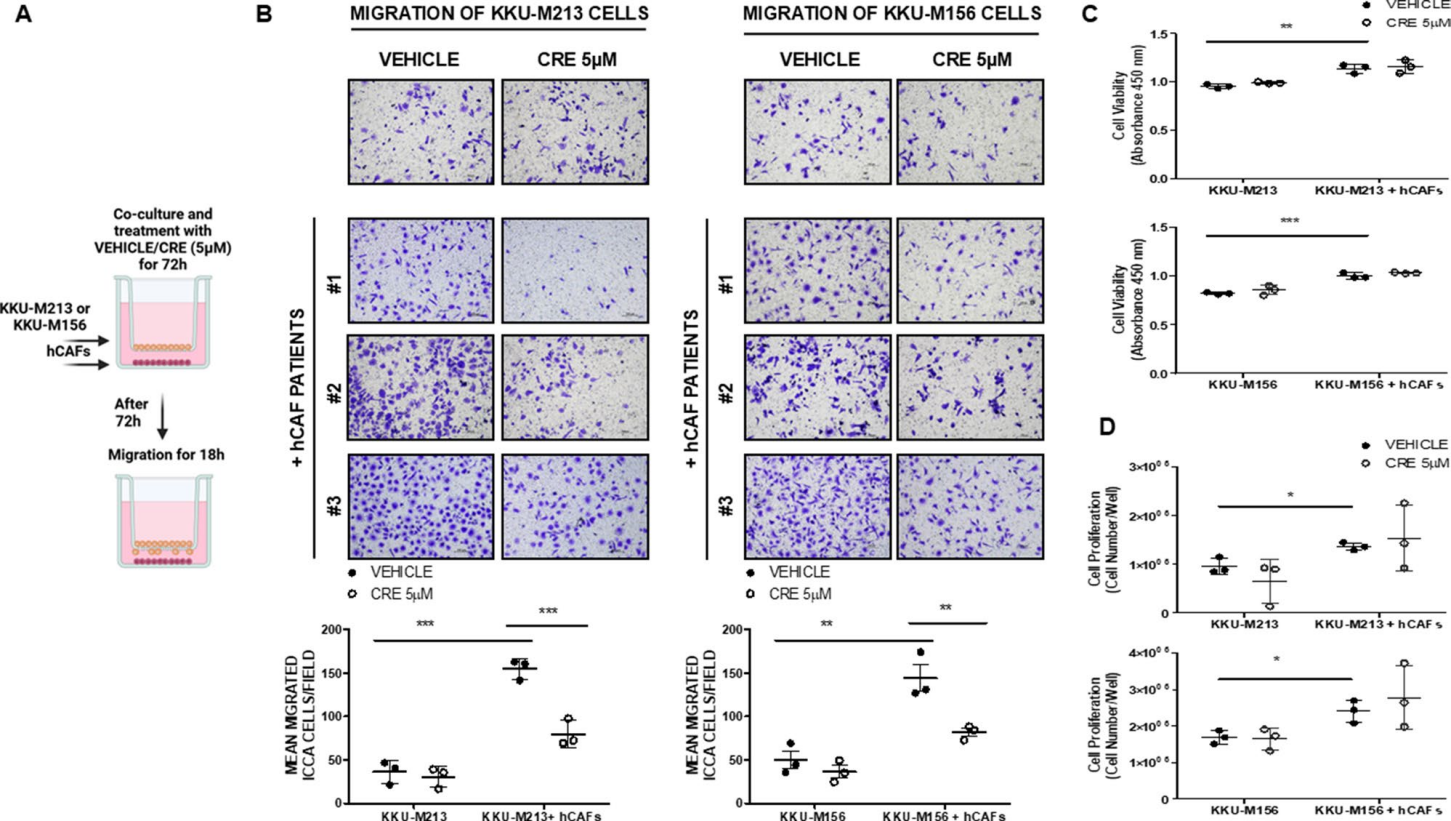
Crenigacestat effectiveness on hCAFs/iCCA cells cross-talk migration. (**A**). Schematic representation of the experimental model of KKU-M213 and KKU-M156 migration without and with hCAFs treated with Crenigacestat (5 µM). (**B**). Transwell migration assays showed that Crenigacestat significantly reduced the migration of **A**) KKU-M213 and **B**) KKU-M156 cells in co-culture with three hCAFs (#1, #2, #3) compared to the vehicle. The number of migrated cells was quantified in five random microscope fields for each treatment in experiments performed three times with three hCAFs. (10× magnification, scale bar 100 μm). (**C**). KKU-M213 and KKU-M156 cell viability treated with Crenigacestat (5 µM) was evaluated with the CyQUANT™ XTT Cell Viability kit. Crenigacestat did not affect the viability of both iCCA cells in culture with or without hCAFs. (**D**). The KKU-M213 and KKU-M156 cell proliferation was evaluated with Trypan Blue. Crenigacestat did not significantly influence the number of both iCCA cells in culture with or without hCAFs. Values are presented as mean ± SD. **p < 0.01 and ***p < 0.001

To investigate the effectiveness of Crenigacestat on the hCAFs/iCCA cells cross-talk, we treated each cell component individually, and then combined them for challenging iCCA cells to migrate. Firstly, we treated both iCCA cell lines with Crenigacestat or vehicle under the same experimental conditions previously described; we seeded them in the upper part of the insert coated at the bottom part with Coll I, and challenged them to migrate for 18 h in fresh medium without any drug but in the presence of hCAFs seeded at the bottom part of the transwell. As reported in Supplementary Fig. 1, the treatment with Crenigacestat did not affect cell migration of both iCCA cell lines as compared to the treatment with vehicle. Secondly, in a different set of experiments, we treated hCAFs cultured at the bottom part of the transwell with Crenigacestat or vehicle under the same experimental conditions previously described, and challenged both iCCA cell lines seeded on the top of the insert coated at the bottom part with Coll I to migrate for 18 h in the presence of fresh medium without any drug. As reported in Supplementary Fig. 2, also in this case Crenigacestat did not affect cell migration as compared to vehicle. Thirdly, in a further set of experiments, we treated separately both iCCA cell lines and hCAFs with Crenigacestat and vehicle in different wells, then we allowed iCCA cells lines to migrate for 18 h on filters coated with Coll I in the absence of any drug but in the presence of hCAFs, previously seeded. As shown in Supplementary Fig. 3, also in this case Crenigacestat treatment did not affect cell migration. Finally, we treated cells for 72 h with Crenigacestat or vehicle KKU-M213 as well as KKU-M156 cells cultured on the top of the insert and hCAFs at the bottom of the transwell at the same time. Then, iCCA cells were trypsinized, washed and seeded on the top of a new insert coated at the bottom part of the filter with Coll I, and challenged to migrate for 18 h in fresh medium without any drug, in the presence of the previously treated hCAFs. As reported in Fig. 2A-B, Crenigacestat significantly inhibited KKU-M213 and KKU-M156 cell migration (*p* < 0.001 and *p* < 0.01, respectively) as compared to vehicle, but it did not affect cell viability and proliferation using all the same hCAF preparation in 2D models (Fig. 2C-D). All the experimental conditions were run in experimental triplicate, and each experiment was re peated in biological triplicate using preparations of hCAFs isolated from three different patients who underwent surgery for iCCA.

In conclusion, Crenigacestat inhibits iCCA cell migration only when the hCAFs/iCCA cell cross-talk occurs.

### Characterization of hCAFs/ iCCA cells in hetero-spheroid 3D structures

**Fig. 3:**
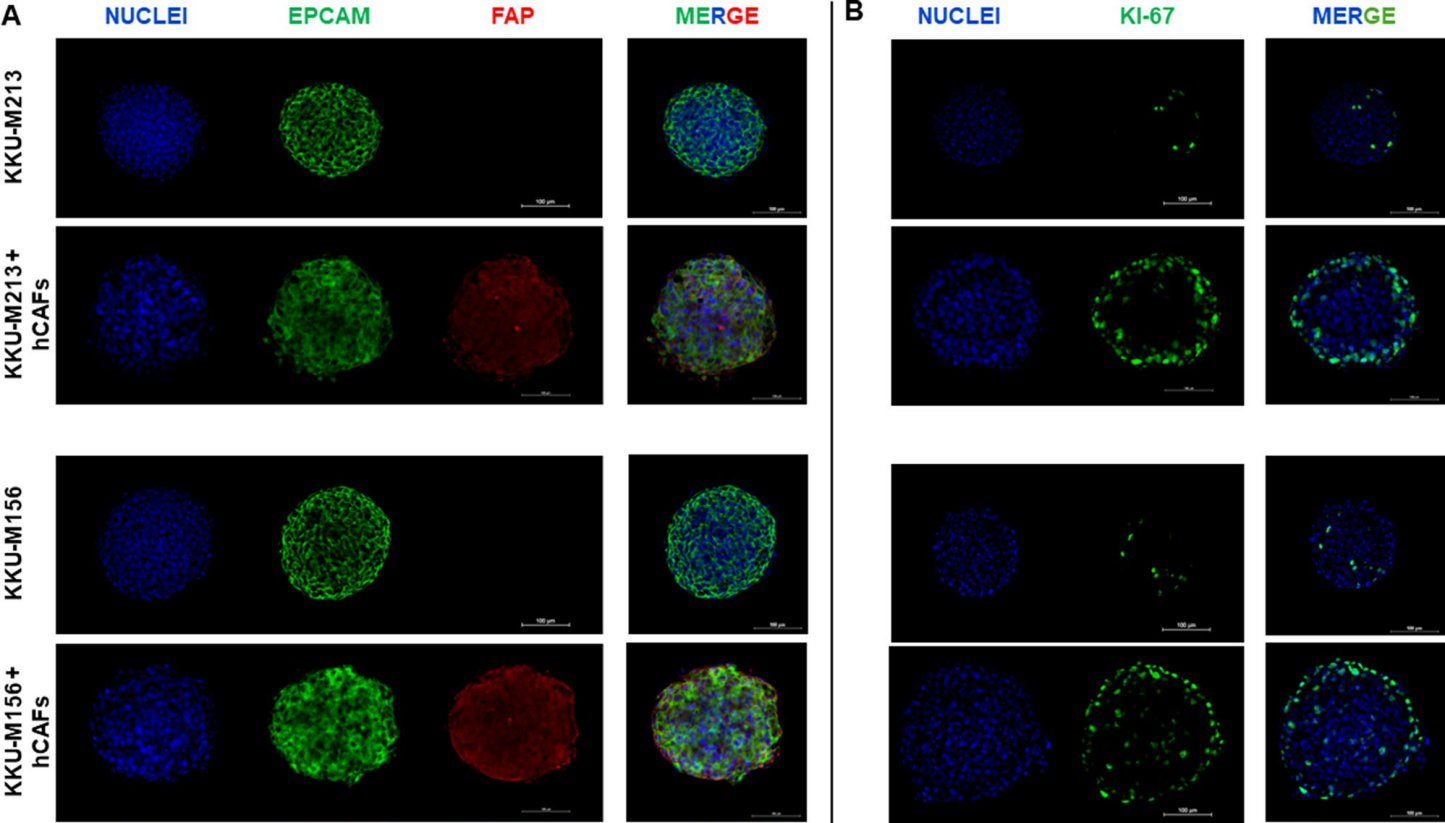
Immunofluorescence characterization of 3D-hCAFs/iCCA spheroids. (**A**). Representative confocal microscopy images of homo- and hetero-spheroids established by KKU-M213 and KKU-M156 alone or in co-culture with hCAFs. The EpCAM marker, considered an epithelial-specific marker, is shown as a green fluorescent signal. The FAP marker, used as a specific marker for fibroblast activation, is shown as a red fluorescent signal. (**B**). Positive staining for Ki-67 indicates cell proliferation and demonstrates that hCAFs influence proliferation in hetero-spheroids. Nuclei are shown as a blue fluorescent signal stained with DAPI. (20× magnification, scale bar 100 μm)

To investigate in further depth the role of the hCAFs/iCCA cells cross-talk we developed a three-dimensional (3D) in vitro model shaped by only iCCA cells (homo-spheroids) or by hCAFs and iCCA cells (hetero-spheroids), in which both cell types are in tight contact. Briefly, 3D-structures formed exclusively by iCCA cells or by both hCAF and iCCA cells were characterized by immunophenotyping using Epithelial Cell Adhesion Molecule (EpCAM) and Fibroblast Activation Protein (FAP) expression, as well-known markers of iCCA and hCAFs cells, respectively. As expected, immunofluorescence staining revealed EpCAM-positive expression in homo- and hetero-spheroids of KKU-M213 and KKU-M156, while FAP-positive expression was positive only in hetero-spheroids, Fig. 3A and movies (Supplementary Video 1–4), thus confirming the presence of both iCCA cells and hCAFs. Then, homo and hetero-spheroids were stained with anti-Ki-67 antibodies, to explore cell proliferation activity. As described in Fig. 3B and movies (Supplementary Video 5–8), the number of Ki-67 positively stained cells was significantly (*p* < 0.001) higher in hetero-spheroids, 52.4% and 48.1% respectively for the presence of KKU-M213 and KKU-M156 with hCAFs versus 6.8% and 11.0% for the corresponding homo-spheroids.

In conclusion, a cross-talk between hCAFs/iCCA cells increased proliferation.

### Crenigacestat inhibits the viability and invasion of hetero-spheroids

**Fig. 4:**
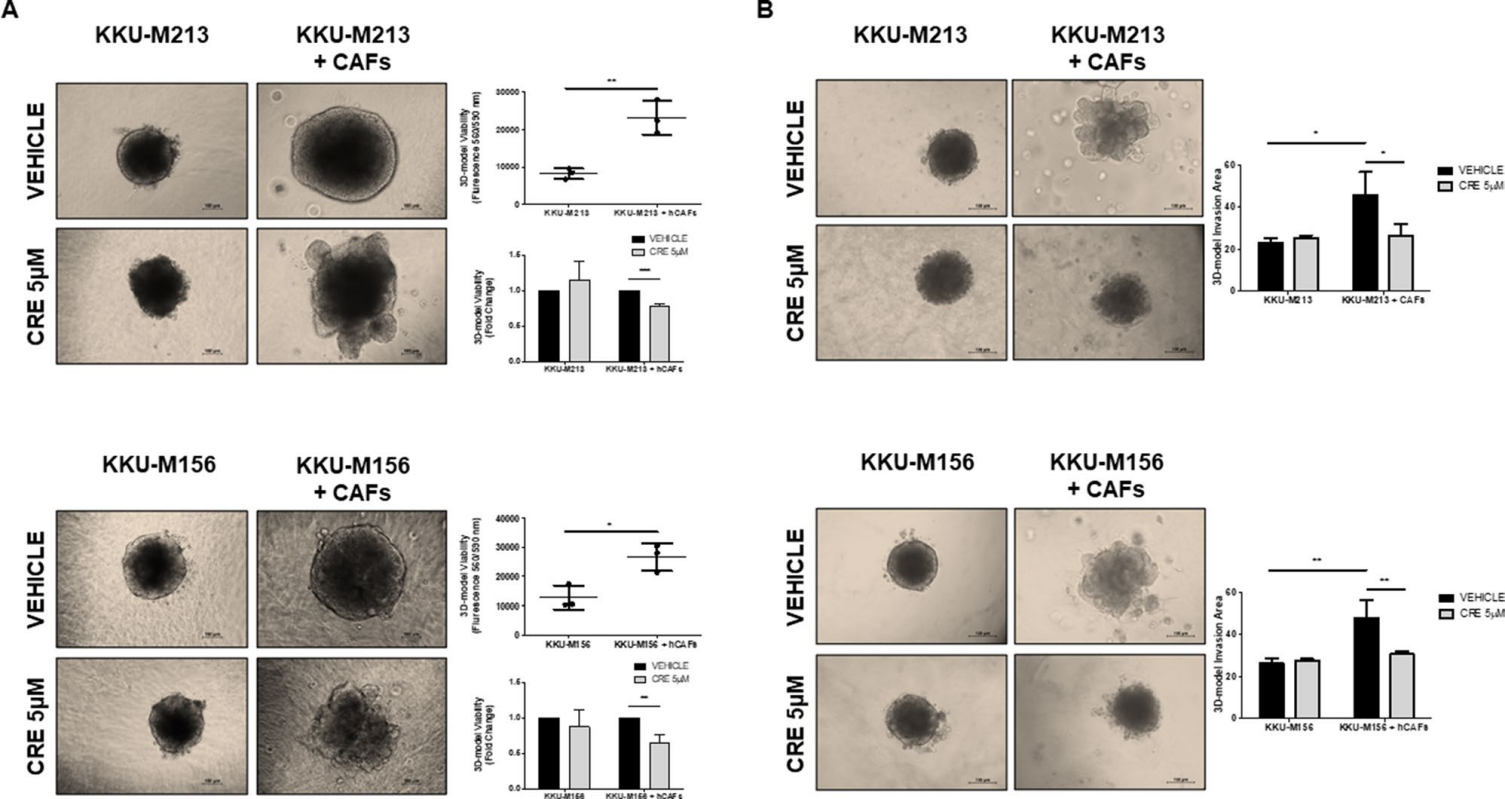
Effect of Crenigacestat on hCAFs/iCCA cell spheroids viability and invasion assay. The composite hydrogels formed a 3D environment that allows **A**) the viability or **B**) invasion of cells. hCAFs promote growth, viability, invasion and migration of KKU-M213 and KKU-M156 hetero-spheroids compared to homo-spheroids. Crenigacestat treatment inhibited viability, invasion and migration of KKU-M213 and KKU-M156 spheroids co-cultured with hCAFs. (10× magnification, scale bar 100 μm). Experiments were performed three times with three hCAFs. Values are presented as mean ± SD. *p < 0.05; **p < 0.01 and ***p < 0.001

To investigate the effectiveness of Crenigacestat on the iCCA/hCAFs cross-talk modulating cellular viability and invasion in 3-D models, we bioengineered two distinct types of biological scaffolds formed by different extracellular matrix proteins enriched with hydrogel to better support cell viability or cell invasion, respectively. Firstly, we cultured, for 96 h, KKU-M213 or KKU-M156 hetero-spheroids embedded in the proliferative permissive hydrogel, whereby significantly (*p* < 0.01 and *p* < 0.01, respectively), they doubled the surface area and showed a significantly (*p* < 0.01 and *p* < 0.05, respectively) increased viability compared to the homo-spheroids, see Supplementary Fig. 4 and Fig. 4A. Under the same experimental conditions previously described, we tested homo- and hetero-spheroids with Crenigacestat, and after 96 h, the drug treatment significantly reduced KKU-M213 and KKU-M156 hetero-spheroids viability (*p* < 0.001 and *p* < 0.01 respectively) as compared to vehicle. Consistently with the results described above, Crenigacestat treatment did not affect homo-spheroids viability (Fig. 4A).

Next, we cultured homo- and hetero-spheroids in invasion-permissive hydrogel for 72 h. As observed in Fig. 4B, KKU-M213 and KKU-M156 hetero-spheroids significantly invaded the surrounding area (*p* < 0.05 and *p* < 0.01, respectively) as compared to homo-spheroids. Furthermore, Crenigacestat treatment significantly inhibited the invasion of KKU-M213 and KKU-M156 hetero-spheroids (*p* < 0.05 and *p* < 0.01, respectively), but not of homo-spheroids, Fig. 4B. All the experiments of 3D model viability and invasion were performed in technical triplicate and in biological triplicate using preparations of hCAFs isolated from three different iCCA patients.

In conclusion, this bulk of data suggests that the iCCA/hCAFs crosstalk plays a key role in the growth and invasion of hetero-spheroids. Consistently, Crenigacestat reduces cell viability and invasion only in the presence of the iCCA/hCAFs crosstalk.

### Crenigacestat reduces the fibrosis and growth of hCAFs/iCCA tumors in vivo

**Fig. 5:**
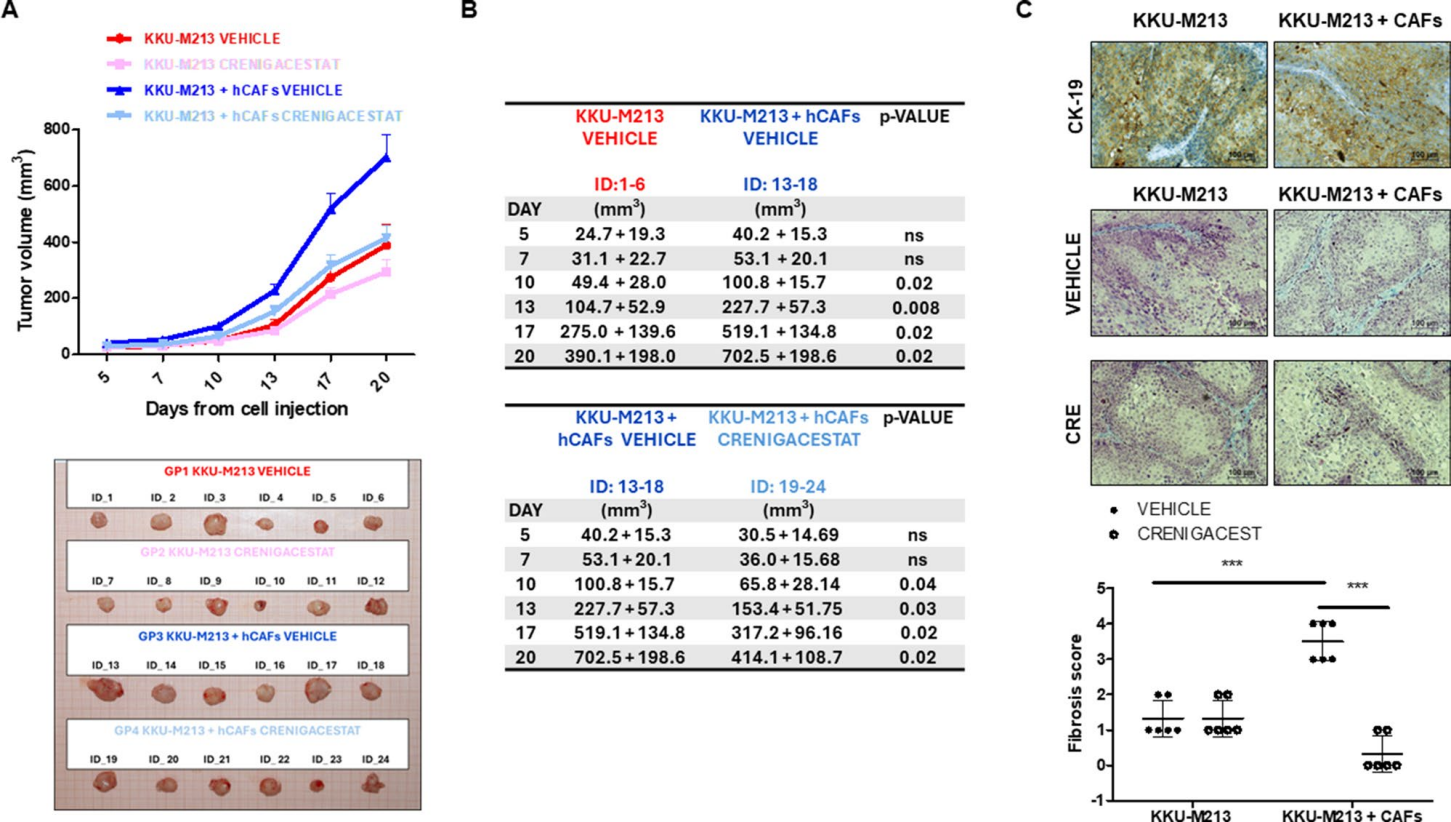
Crenigacestat reduced iCCA progression promoted by hCAFs/iCCA cells cross-talk in the xenograft model. (**A-B**). The tumor volumes were evaluated in nude mice co-injected with KKU-M213 and hCAFs cells. Only KKU-M213 cells were injected into the control group. hCAFs that were co-injected with iCCA cells enhanced tumor growth. Crenigacestat significantly reduced the tumor progression induced by hCAFs, compared with the control (vehicle). Data are presented as the mean ± SD. *N* = 6 mice per group; *p < 0.05, p-values were obtained using the Mann-Whitney U Test. (**C**). Immunohistochemical staining showed a similar CK-19 expression pattern in both tumor models (upper panel). Masson’s trichrome staining shows different amounts of intercellular collagenous matrix in the two models, with and without Crenigacestat treatment (lower panel). An adapted METAVIR was used to quantify the fibrosis in treated and untreated mice, as reported in the graphs. The staining quantification was calculated as the mean intensity staining of the whole section from the KKU-M213 and KKU-M213/hCAFs tumors of each mouse treated with Crenigacestat compared to vehicle

To better investigate the hCAFs/iCCA cells cross-talk in vivo, we generated a xenograft model by injecting KKU-M213 exclusively or in combination with hCAFs but normalizing for the total number of cells. Tumors originated by KKU-M213 and hCAFs grew larger as compared with those originated by KKU-M213 alone, and this difference was already significant (*p* < 0.02) after 10 days, and persisted until the end of the experiment. Consistently with all the in vitro results, Crenigacestat significantly (*p* < 0.05) reduced the volume of the hCAFs/KKU-M213 tumors after 10 days of treatment and maintained this effect until the end of treatment, showing an even more significant difference (*p* < 0.02) when compared to untreated mice. On the contrary, no statistically significant effects were observed on tumors generated by KKU-M213 alone during the whole treatment (Fig. 5A-B).

To confirm that our xenograft model reliably resembles a human iCCA, we stained sections from KKU-M213 and KKU-M213/hCAFs tumors with an anti-CK-19 antibody. As reported in Fig. 5C, CK-19, a specific marker of CCA, was diffusely expressed, showing a membranous and cytoplasmic homogenous pattern. A smaller but still relevant number of tumor cells displayed consistent cytokeratin 7 immunoreactivity (data not shown). Following Masson trichrome staining, all sections showed variable amounts of intercellular collagenous matrix. In KKU-M213 tumors, it appeared as thin light blue bundles surrounding the periphery of tumor cell clusters, and including scarce and sparse fibroblasts, lympho-monocytes, and neutrophilic granulocytes. No changes were observed after Crenigacestat treatment. On the contrary, in KKU-M213/hCAFs tumors the extracellular collagen bundles were much larger, more intensely stained in blue, and included more numerous fibroblasts, which were also more tightly packed. After treatment with Crenigacestat, collagen bundles were less evident around the tumor clusters and they showed a lighter bluish discoloration and included smaller amounts of fibroblasts inside. Furthermore, we quantified the amount of the deposited fibrotic matrix using an adapted METAVIR score, as previously reported [22]. Liver fibrosis was significantly (*p* < 0.001) more abundant in KKU-M213/hCAFs than in KKU-M213 tumors, where it was organized as reactive tumoral stroma. Crenigacestat treatment significantly (*p* < 0.001) reduced liver fibrosis on KKU-M213/hCAFs compared to KKU-M213 tumors, but it did not display any effect on other models, Fig. 5C.

In conclusion, the co-injection of both hCAFs and KKU-M213 cells forms bigger tumors, but they are druggable with Crenigacestat.

### Transcriptomic analysis of explanted tumor masses

**Fig. 6:**
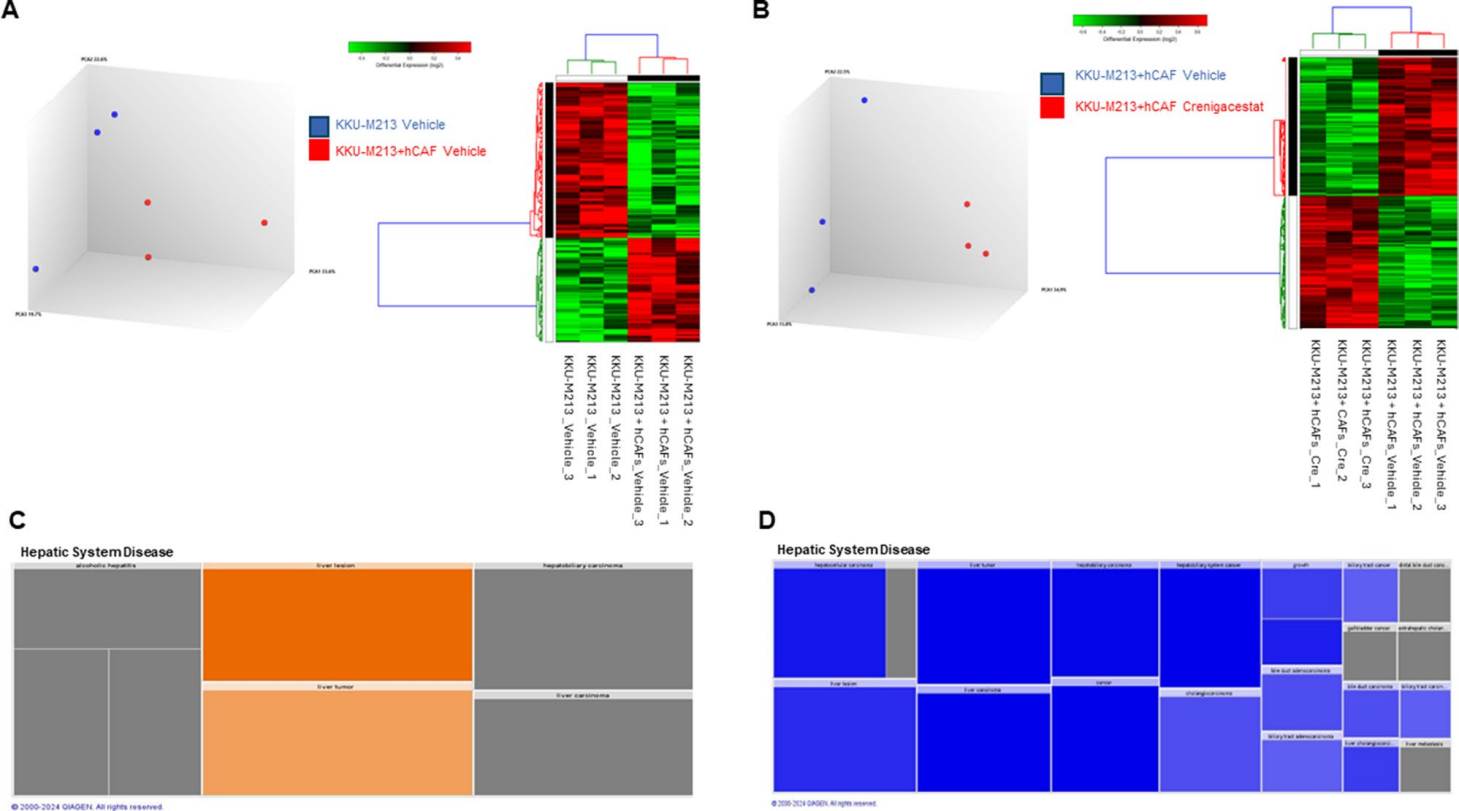
Transcriptomic profiling of explanted tumor masses. (**A**) PCA and hierarchical clustering heatmap using DEGs shows a clear separation of masses explanted from the xenograft model derived from co-injection of KKU-M213 and hCAFs with those derived from only KKU-M213. (**B**) PCA and the hierarchical clustering heatmap using DEGs showed a clear separation of masses explanted from co-injection of KKU-M213 and hCAFs with and without Crenigacestat treatment. Each row represents a gene, and each column represents a sample. The expression levels of genes are indicated by the color bar above the heatmap. Increased expression is shown in red whereas decreased expression is shown in green. IPA results using the annotations “Disease & Function” revealed that most genes were involved in Hepatic System Diseases in the comparison between KKU-M213 vehicle and hCAFs vs. KKU-M213 vehicle (**C**) and in the comparison of KKU-M213 + hCAF vehicle vs. KKU-M213 + hCAF Crenigacestat vs. KKU-M213 + hCAF vehicle (**D**). Results are visualized as a hierarchical heatmap where the boxes represent a category of related functions. Each colored rectangle represents a particular biological function or disease, and the color indicates the state of prediction: increase (orange) and decrease (blue)

To investigate the molecular mechanisms triggered by the hCAF/KKU-M213 cross-talk in the xenograft mice models with and without Crenigacestat treatment, we performed a transcriptomic analysis on tissues explanted from xenograft mice (*n* = 3/group). Firstly, we compared the tumor masses derived from co-injected KKU-M213 and hCAFs with those derived from only KKU-M213. This comparison identified 129 DEGs between the two groups, and the expression profiles of these DEGs were visualized through principal component analysis (PCA) and the hierarchical clustering heatmap (Fig. 6A). Then, we verified the molecular changes induced by Crenigacestat in the xenograft model derived from co-injected KKU-M213 and hCAFs, and from KKU-M213 only. We detected 554 genes differentially expressed between the two groups. PCA and hierarchical clustering heatmap showed a clear separation between the two groups (Fig. 6B).

**Fig. 7:**
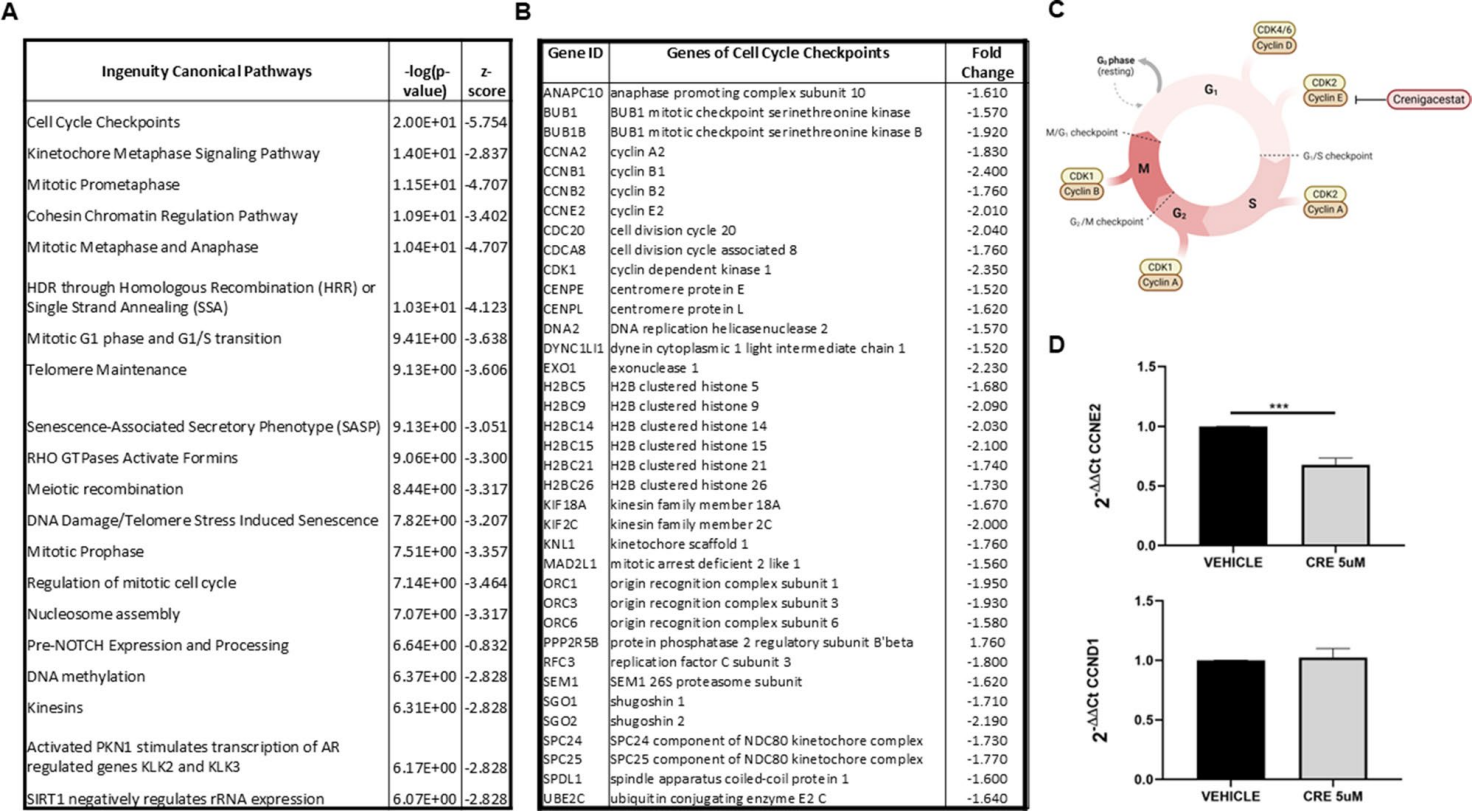
Crenigacestat affected in vivo tumor growth, inhibiting CCNE2E2 cyclin. (**A**). Top 20 significant pathways from DEGs involved in the reduction of liver lesions after Crenigacestat treatment in the xenograft model derived from co-injection of KKU-M213 with hCAFs. (**B**). List of 37 DEGs modulated by Crenigacestat and involved in the cell cycle checkpoints. (**C**). Schematic representation of Crenigacestat modulation of CCNE2 expression and downstream effects on cell-cycle progression. (**D**). CCNE2 and CCND1 mRNA expression levels on masses of the xenograft model derived from co-injection of KKU-M213 and hCAFs, by quantitative real-time PCR. ***p < 0.001

To gain insight into the biological effects of DEGs, we performed an IPA core analysis. The IPA annotations “Disease & Function” revealed that the most numerous genes for both comparisons were present in Hepatic System Diseases. In particular, based on this analysis, genes from co-injected hCAFs and KKU-M213 tumors were able to increase the formation of Liver lesions and Liver tumors (Fig. 6C). Contrariwise, the treatment of Crenigacestat in this mice model reduced the formation of Liver lesions and Liver tumors. Other biological functions affected by Crenigacestat were Hepatobiliary carcinoma, Hepatobiliary system cancer, Cholangiocarcinoma, Liver cholangiocarcinoma and Growth (Fig. 6D). Next, we evaluated the modulated canonical pathways that led to a reduction of liver lesions after Crenigacestat treatment in the xenograft model derived from co-injected KKU-M213 and hCAFs. We focused our attention on the genes that are involved in the function Liver lesions, which numbered 280 of the total 554, and we found that in the top 20 modulated pathways, several signaling pathways controlled the cell cycle progression (Fig. 7A).

Specifically, 37 differentially expressed genes (DEGs) were modulated by Crenigacestat and regulated the cell cycle checkpoints (Fig. 7B). Of note, several genes that encode for cyclins (CCNA2, CCNB1, CCNB2, CCNE2) were downregulated after Crenigacestat treatment. Among these genes, we hypothesized that Crenigacestat affected CCNE2 expression that, in turn, reduced the expression of downstream cyclins like CCNA2, CCNB1, CCNB2 (Fig. 7C). To validate the sequencing results, we performed quantitative real-time PCR for CCNE2 on masses of the xenograft model derived from co-injection of KKU-M213 and hCAFs. We found that, after Crenigacestat treatment, the mRNA expression of CCNE2 significantly decreased compared to untreated masses (*p* < 0.001, Fig. 7D) suggesting that cell cycle progression could be arrested in the G1 phase. As control, we also verified the expression of CCND1 that controls the previous phase of cell cycle and we observed that Crenigacestat treatment did not have any influence on its expression (Fig. 7D).

In conclusion, IPA core analysis revealed that genes from tumors with a hCAFs/KKU-M213 cross-talk had increased liver lesions, and that this was decreased by Crenigacestat treatment. Finally, cell cycle progression was the most notably modulated pathway following Crenigacestat treatment.

### Crenigacestat arrests the cell cycle at the G0/G1 phase in hetero-spheroids

**Fig. 8:**
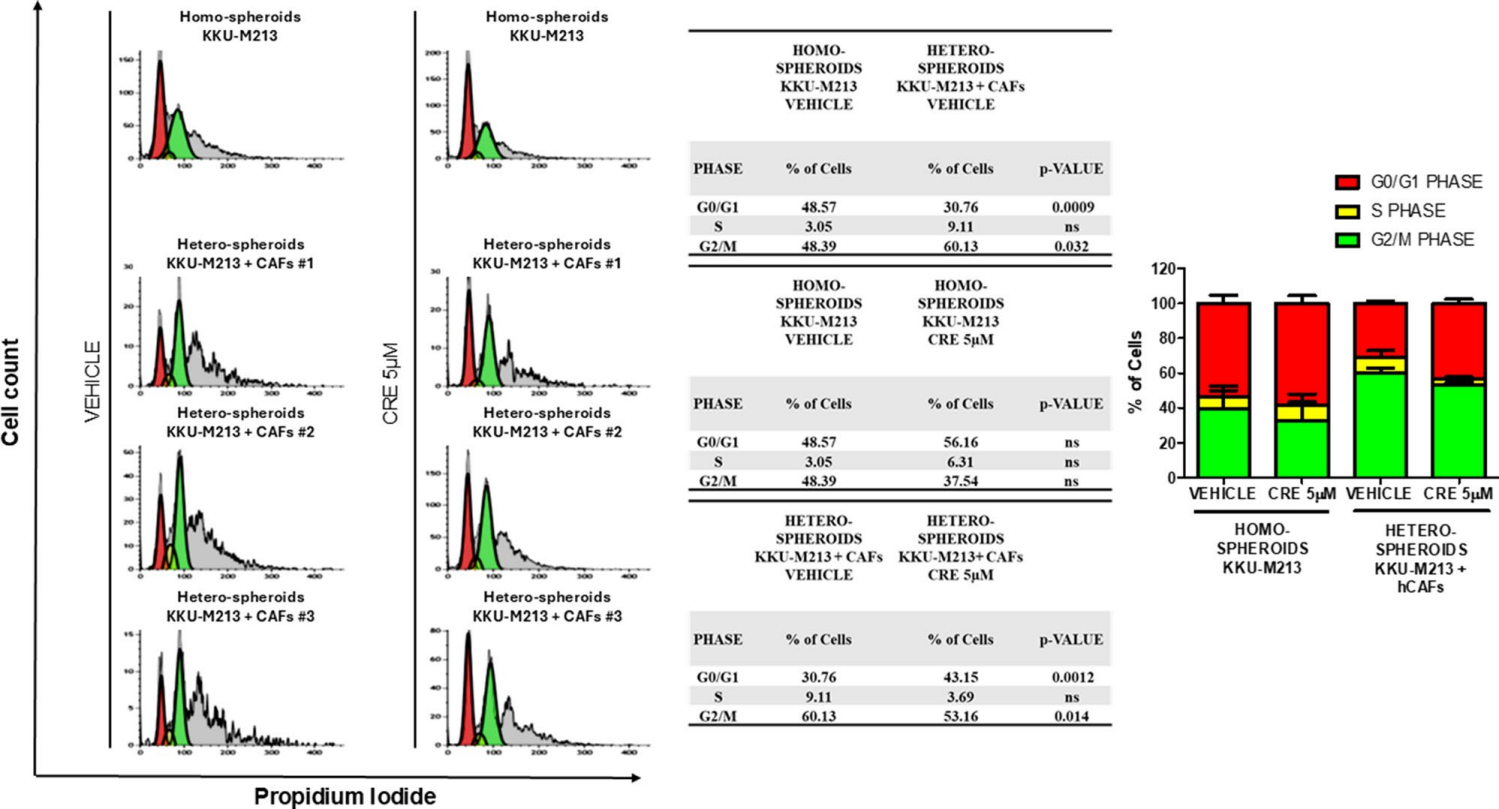
Crenigacestat induced hetero-spheroids cell cycle arrest in the G0/G1 phase. A. Cell cycle distribution of KKU-M213 and KKU-M213 with hCAFs spheroids treated with vehicle or Crenigacestat for 96 h was examined by flow cytometry. An increased G2/M cell cycle phase was observed in hetero-spheroids compared to homo-spheroids and Crenigacestat treatment induced a G0/G1 cell cycle arrest in hetero-spheroids compared to vehicle. The calculated percentage of cell cycle distribution was presented as mean ± SD from three independent experiments

To validate the transcriptomic and bioinformatic results previously described, we investigated Crenigacestat effectiveness on cell cycle homo and hetero-spheroids. Analyzed by flow cytometry, consistently with all previous data, the hCAFs/iCCA cells cross-talk significantly (*p* < 0.05) increased the cell numbers in the G2/M phase compared to the homo-spheroids, leading to a greater growth of hetero-spheroids. Next, both homo and hetero-spheroids were treated with Crenigacestat 5 µM for 96 h. As reported in Fig. 8, Crenigacestat significantly (*p* < 0.01) arrested the cell population of the hetero-spheroids in the G0/G1 cell cycle phase, reaching 43.15%, while with vehicle treatment it reached 30.76%. Consistently, the G2/M phase in hetero-spheroids significantly (*p* < 0.05) decreased to 53.16% after treatment with Crenigacestat, while after vehicle treatment it reached 60.13%. Finally, Crenigacestat did not display any effect on homo-spheroids. All the experiments were run in triplicate using three different preparations of hCAFs isolated from distinct iCCA patients who underwent surgery.

In conclusion, the hCAFs/iCCA cells cross-talk increased the growth of hetero-spheroids, increasing cells in the G2/M cell cycle phase, while Crenigacestat treatment decreased the growth of hetero- but not homo-spheroids, inducing a decrease of the number of cells in the G2/M cell cycle phase.

## Methods

### Cell lines and reagents

KKU-M213 and KKU-M156 human iCCA cell lines, purchased from the Japanese Collection of Research Bioresources (JCRB) or the American Type Culture Collection (ATCC), were used for the experiments. Cell Lines Service (Eppelheim, Germany) performed cell line authentication. Both cell lines were grown in Dulbecco’s modified Eagle medium (DMEM), (Gibco, Grand Island, NY, USA) supplemented with 10% Fetal Bovine Serum (FBS), Antibiotic-Antimycotic, Sodium Pyruvate and Hepes, (Gibco, Grand Island, NY, USA). Cells were cultured at 37 °C in a 5% CO_2_ humidified atmosphere. Cell lines, before use, were tested mycoplasma-free using the MycoFluor™ Mycoplasma detection Kit (ThermoFisher Scientific, Waltham, MA, USA). Crenigacestat (LY3039478, Selleckchem Chemicals, Houston, TX, USA) was used to treat iCCA cell lines in vitro and in animal models in vivo. Stock solutions were prepared in Dimethylsulfoxide (DMSO) (ThermoFisher Scientific, Waltham, MA, USA) and aliquots were stored at -80 °C.

### Establishment of iCCA hCAF culture from fresh human iCCA tissues

Approval of the study was granted by the local Ethics Committee, Istituto Tumori “Giovanni Paolo II” (Bari, Italy) (protocol number: 145; date of release: March 2022); in compliance with the Helsinki Declaration. Informed consent was obtained from all individuals. After surgical resection, iCCA tissue specimens were immediately stored in MACS tissue storage solution (Miltenyi Biotec, Bergisch Gladbach, Germany) and processed for hCAFs isolation as previously reported [23]. iCCA hCAFs were isolated from three patients. We performed enzymatic and mechanical digestion of iCCA tissue fragments in HBSS solution with 50–200 U/mL collagenase Type IV (Thermo Fisher Scientific, Waltham, MA, USA), 3 mM CaCl_2_, and Antibiotic–Antimycotic (Thermo Fisher Scientific, Milan, Italy) at 37 °C by rotation for 2 h or more as needed. The resulting cells were harvested by recovering the volume of digestion and washed with PBS. The cell population harvested was cultured in complete IMDM (Iscove’s Modified Dulbecco’s Medium) with 20% FBS and Antibiotic–Antimycotic at 37 °C in a 5% CO_2_ humidified atmosphere to obtain hCAF cultures.

### Viability and proliferation assay on iCCA cell in co-culture with hCAFs

For the co-culture, 15 × 10^3^ iCCA cells were seeded onto 24-well plates with complete DMEM medium. Additionally, 20 × 10^3^ hCAFs were seeded onto transwell inserts with 0.4 μm pore size (Corning, Bedford, MA, USA) in complete IMDM medium. After 24 h, the transwell inserts with seeded hCAFs were transferred to 24-well plates for co-culturing with iCCA cells. As control, iCCA cells without hCAFs in transwell inserts were prepared. iCCA cell cultures and hCAFs/iCCA co-cultures prepared in this way were treated with vehicle and Crenigacestat (5 µM) in IMDM + 1% FBS for 72 h. At the end of treatment, iCCA cell viability in the presence or absence of hCAFs was determined with the CyQUANT™ XTT Cell Viability kit (ThermoFisher Scientific, Waltham, MA, USA) and for proliferation by Trypan Blue cell counting. Data are expressed as the mean ± SD of three independent experiments with three hCAFs.

### Transwell migration assay

KKU-M213 or KKU-M156 cell migration was induced for 18 h, after 72 h of treatment. In detail, we developed three different co-culture approaches: pre-treating for 72 h 15 × 10^3^ iCCA cells onto transwell inserts with 0.4 μm pore size or 40 × 10^3^ hCAFs onto 24-well plates separately, or both cell types simultaneously in co-culture with Crenigacestat (5 µM) or vehicle. In each case, after the treatment, we evaluated the iCCA cell migration in co-culture with 40 × 10^3^ hCAFs. As described for the vitality assay, also for the study of this cell functional aspect, we used a control condition, namely the migration of iCCA cells without hCAFs seeded in the bottom of the wells. Cell migration was allowed by using transwell inserts suitable for 24-well plates, with 6.5 mm internal diameter, and 8 μm pore size (Corning, NY, USA). The membrane of these transwell inserts had been coated with rat tail collagen I (final concentration 10 µg/mL) on the lower surface for 2 h at room temperature. 20 × 10^3^ iCCA cells were suspended in 200 µL of serum-free IMDM medium and loaded onto the top chamber of transwell inserts. Cells were allowed to migrate for 18 h in IMDM medium + 10% FBS at 37 °C and 5% CO_2_. After incubation, the migrated cells were fixed in 4% PFA (pH 7.2 in PBS) and stained with crystal violet for 10 min. Five fields per membrane were captured in bright field at 10× magnification. The analysis was performed on the mean number of migrated cells/field. Data are expressed as the mean ± SD of three independent experiments with three hCAFs.

### 3D homo-spheroid and hetero-spheroid cultures

Three-dimensional (3D) cell culture models were set up using the hanging drop method as previously described [24]. Briefly, cells were suspended, at a concentration of 1 × 10^5^ cells/ml, in medium with 0.24% methylcellulose (Sigma, St. Louis, MO, USA). Forty drops of 25 µl each were pipetted onto the lid of 100 mm dishes. In this way each drop and thus each spheroid consisted of 2.5 × 10^3^ cells. Homo-spheroids were developed starting from a single cell type, KKU-M213 or KKU-M156 cells. Instead, hetero-spheroids were developed by respecting the 1:3 rate for KKU-M213 or KKU-M156 cells: hCAFs. After 3 days of incubation at 37 °C and 5% CO_2_, spheroids were transferred into different culture supports depending on the cell function assay. Each experimental condition was performed three times and hetero-spheroids were replicated with three hCAFs. Values are presented as mean ± SD.

### Flow Cytometric analysis of cell cycle

For cell cycle analysis, the recovered spheroids were cultured on an ultralow-attachment culture plate (Corning, Bedford, MA, USA) and treated with vehicle or Crenigacestat (5µM) twice for 5 days. The cell cycle was induced on cell suspensions obtained after disruption of the homo- and hetero-spheroids. Briefly, floating spheroids were disrupted using Tryple™ Select (Gibco, life Technologies corporation, NY, USA) plus 1mM EDTA pH 8.0 (Invitrogen by ThermoFisher Scientific, USA) and pipetting every 10 min. 1 × 10^6^ cells were washed once in cold PBS and fixed with cold 70% ethanol at 4 °C overnight. The fixed cells were then washed twice in PBS, the supernatant was discarded, and the cells were treated with of RNase A (Sigma, United States; 100 µg/mL) for 15 min at 37 °C. Propidium iodide; Sigma, United States; 200 µL of 50 µg/mL stock) was then added to the cells and incubated for 30 min at 4 °C in the dark. The DNA content of the cells was determined by flow cytometry.

### Spheroid viability assay

For the viability assay, the spheroids were embedded in engineered hydrogel networks made of bovine skin hydrolyzed collagen and Corning^®^ Matrigel^®^ Matrix; the pH was neutralized with 0.5 M acetic acid. To prevent the spheroids from settling and sticking to the bottom of the well, a drop of matrix was placed in the well and allowed to dry for 20 min before embedding the samples into the matrix. In addition, spheroids were treated with vehicle and Crenigacestat (5µM). After 5 days of treatment, cell viability was assayed using the CellTiter-Blue™ Viability Assay (Promega, Tokyo, Japan).

### Spheroid invasion assay

The spheroids were embedded in an engineered hydrogel network. The bovine collagen type I solution and Corning^®^ Matrigel^®^ were mixed and neutralized with 0.5 M acetic acid to obtain a matrix suitable for the invasion assay. Fresh medium was added to the final gel solution, to simulate cell embedding. Spheroids were treated with Crenigacestat (5µM) or vehicle for 72 h. Images were captured in bright field at 10× magnification. The analysis was performed on the area of invasion ratio of treated spheroids/untreated spheroids.

### Spheroid immunofluorescence

For immunofluorescence staining, the spheroids were fixed in a 4% PFA solution at 4 °C for 30 min, and then washed twice in 0.1% Triton X-100 in TBS. After washing, samples were permeabilized with 0.5% Triton X-100 in TBS at room temperature. Then, the spheroids were incubated with anti-FAP (1:150, Abcam, Cambridge, UK), anti-EPCAM antibodies (1:800, Cell Signaling Technologies, MA, USA) in an antibody dilution buffer (2% bovine serum albumin/0,1% Triton X-100 in TBS solution) and anti-Ki67 (1:250, Abcam, Cambridge, UK), overnight at 4 °C in rotation. The following day, after washing, samples were incubated with secondary goat anti-rabbit immunoglobulin G H&L (1:500 Alexa Fluor 594, Thermo Fisher Scientific, Waltham, MA, USA) for FAP, with secondary goat anti-mouse immunoglobulin G H&L (1:50 Alexa Fluor 488, Thermo Fisher Scientific, Waltham, MA, USA) for EPCAM and with secondary goat anti-rabbit immunoglobulin G H&L (1:50 Alexa Fluor 488, Thermo Fisher Scientific, Waltham, MA, USA) for Ki67. The incubation with secondary antibodies lasted 3 h at room temperature in the dark. After further washing, nuclei staining was performed with 0.5 ng/ml of PureBlu DAPI Nuclear Staining Dye (Bio-Rad Laboratories, USA) in 0.1% Triton X-100 in TBS, incubating for 1 h at room temperature in the dark. Spheroids were put on the slides and covered with ProLong™ Diamond antifade mounting medium (Invitrogen by ThermoFisher Scientific, USA). Confocal images and movies of spheroids were acquired using the Nikon Ti2-E Inverted Research Microscope equipped for confocal imaging in conjunction with a Nikon A1rSi Laser Point Scanning Confocal System, Plan Fluor Ph 20X objective and NIS-Elements “AR” 5.0 software and improved by deconvolution method based on Richardson-Lucy Algorithm.

### In vivo study

Housing and all procedures involving the mice were performed according to the protocol approved by the Ethics Committee (Protocol number 257/2023-PR, date of release 29/03/2023) at Biogem Animal House in Ariano Irpino (Avellino, Italy) following the National Academy of Sciences Guidelines. Two million KKU-M213 cell lines or KKU-M213:hCAFs, respecting the 1:1 ratio, were subcutaneously injected into the flanks of 4–5-week-old female CD1 nude mice. Drinking water was supplied ad libitum. Each mouse was offered a complete daily pellet diet (GLP 4RF21, Mucedola) throughout the study. The analytical certificates of animal food and water were retained at Biogem premises. Each mouse was monitored daily for clinical signs and mortality, and body weight was recorded twice a week. The tumor volume was monitored weekly by a caliper and evaluated with the formula (mm^3^) = [length (mm) × width (mm)^2^]/2, where width and length are the shortest and longest diameters. When the tumor masses volume reached approximately 70–100 mm^3^, the mice were randomly subdivided into 2 experimental groups of six animals and administered Crenigacestat (8 mg/kg) or vehicle by oral gavage every 2 days for 20 days. At the end of the study, mice were sacrificed by cervical dislocation, and tumor samples were collected and sectioned for immunohistochemical, hematoxylin-eosin and trichrome staining or for RNA extraction.

### Histological staining method

Tumor specimens were fixed in 4% paraformaldehyde and embedded in paraffin using standard procedures. To analyze the grade of tissue fibrosis Masson’s trichrome staining with the Mallory trichrome acc. McFarlane kit (DIAPATH) was performed, following the manufacturer’s instructions. The degree of fibrosis was classified according to the adapted METAVIR score as previously reported [19]. The images were acquired with the Eclipse Ti2 microscope (Nikon Inc., Melville, NY, USA).

### RNA extraction

Total RNA from explanted tumor masses was isolated with the miRNeasy mini kit (Qiagen, Hilden, Germany) in combination with the TissueLyser homogenizer (Qiagen, Hilden, Germany) according to the manufacturer’s instructions. The RNA concentration was determined with the Qubit™˝ฏ RNA HS Assay kit (Thermo Fisher Scientific, Waltham, MA, USA) on a Qubit Fluorometer (Thermo Fisher Scientific, Waltham, MA, USA). RNA quality was evaluated using the High Sensitivity RNA ScreenTape (Agilent Technologies, Palo Alto, CA, USA) on an Agilent 4200 TapeStation system (Agilent Technologies).

### Whole transcriptome profiling

Total RNA samples were reverse transcribed using the Ion Torrent™ NGS Reverse Transcription Kit (Thermo Fisher Scientific, Waltham, MA, USA) according to the manufacturer’s instructions. Target region amplification was performed using the Ion AmpliSeq Transcriptome Human Gene Expression core panel (Thermo Fisher Scientific, Waltham, MA, USA) on the Ion Chef System. The barcoded libraries were quantified by qPCR with the Ion Library TaqMan Quantitation kit (Thermo Fisher Scientific, Waltham, MA, USA). Finally, libraries were templated onto the Ion Chef and sequenced using a 540 chip on the Ion GeneStudio S5 Prime system (Thermo Fisher Scientific, Waltham, MA, USA).

Sequencing data are available under accession number GSE273905 at the Gene Expression Omnibus (https://www.ncbi.nlm.nih.gov/geo/query/acc.cgi?&acc=GSE273905).

### Quantitative real-time PCR

cDNA was reverse transcribed using the iScript Reverse Transcription Supermix (Bio-Rad Laboratories) according to the manufacturer’s instructions. Quantitative PCR reactions were performed using SsoAdvanced SYBR green (Bio-Rad Laboratories) and the primers sequences for CCNE2 forward, 5-TCAAGACGAAGTAGCCGTTTAC-3′; reverse, 5-TGACATCCTGGGTAGTTTTCCTC-3′, CCND1 forward, 5-GCTGCGAAGTGGAAACCATC-3′; reverse, 5-CCTCCTTCTGCACACATTTGAA-3′, and Hs_GAPDH_1_SG QuantiTect Primer Assay ID: QT00079247 (Qiagen). The CFX96 System (Biorad, Hercules, CA, USA) was used for Real-Time PCR. Comparative real-time PCR was performed in triplicate, including no-template controls. Relative expression was calculated using the 2^−ΔΔCt^ method.

### Bioinformatics and statistical analyses

Ion Torrent Suite Server v5.16.1 (Thermo Fisher Scientific, Waltham, MA, USA) software was used to generate the transcription data as raw read counts using the ampliSeqRNA plugin with default settings. Downstream analyses were performed with Transcriptome Analysis Console 4.0 software (Thermo Fisher Scientific, Waltham, MA, USA). DEGs were identified with the Limma eBayes method using a 1.5 threshold of fold-change and p-value ≤ 0.05. Hierarchical clustering was generated with Alt Analyze 2.1.3 software [25]. Canonical pathways, biological processes and molecular networks associated with DEGs were analyzed with Ingenuity Pathway Analysis (IPA) software (Qiagen, USA). Biological and technical replicates were analyzed with the most appropriate statistical tests (i.e. t test or ANOVA). For in vivo studies, Mann-Whitney U Test was performed. A p-value ≤ 0.05 was considered statistically significant. Statistical analysis and graphs were generated using GraphPad Prism 5.0 software (La Jolla, CA, USA).

## Discussion

The reactive stroma surrounding and embedding the epithelial cancer cells characterizes the histological aspects of iCCA. CAFs secrete the ECM components of the stroma, and orchestrate constant tissue remodeling as a consequence of the further secretion of proteolytic enzymes, cytokines, and growth factors. Consequently, the biochemical composition and biological functions of the stroma are modified, so that cancer cells engage with ECM components, negotiating the expansion of tumor volume and spread at their best convenience [18, 26, 27]. Based on these aspects, hCAFs are considered worldwide as an important component of the tissue microenvironment that directly or indirectly modulates the aggressiveness of cancer cells [28, 29].

Nevertheless, clear cut evidence proving that the hCAFs/cancer cells crosstalk supports the progression of iCCA has never yet been reported. In this study we demonstrate that the growth of iCCA is stimulated by the tumor/stroma crosstalk, that in turn can be targeted with drugs such as Crenigacestat, leading to an inhibition of iCCA progression. We based our conclusion on the following data: (i) iCCA cells migrated and proliferated more efficiently only when in tight contact with hCAFs for a sufficient period to establish a cross-talk, (ii) Crenigacestat selectively inhibited iCCA cell migration stimulated by the cross-talk; (iii) the hetero-spheroids formed by hCAFs and the iCCA cell lines KKU-M213 or KKU-M156, by establishing a cross-talk, proliferated and invaded more efficiently than homo-spheroids formed by only KKU-M213 or KKU-M156 cells; Crenigacestat inhibited the growth and the proliferation of hetero- but not of homo-spheroids; iiii) in xenograft models, tumors generated by co-injection of both hCAFs and KKU-M213 cells are bigger than those generated by KKU-M213, while Crenigacestat inhibited the growth of the former but not of the latter tumors; iiiii) IPA core analysis of the transcriptomic investigation on tumoral masses of co-injected hCAFs and KKU-M213 cells pointed out that genes increased the formation of liver lesions, that was then inhibited by Crenigacestat; iiiiii) Crenigacestat downregulated Cyclin-E2 with a consequent arrest of the cell-cycle checkpoint in hCAF/KKU-M213 tumors, and in heterospheroids it arrested the cell cycle in the G2/M phase.

The novelty of our study is the focus on targeting the tumor-stroma cross-talk, whereas other recent studies highlighted the effects consequent to targeting specific pathways such as hedgehog in pancreatic cancer. In this case, IPI-926 directly targeted hCAFs, affecting the number of ɑ-SMA positive cells, and therefore their capability of secreting ECM components. The study also shows that IPI-926 inhibitor rearranges the tissue microenvironment composition, reducing the deposition of Coll I, the surrounding fibrotic reaction, and increasing microvascular density and the delivery of chemotherapeutic agents [30].

On the other hand, the relevance of an increased prevalence of α-SMA+/CAFs in the stroma of iCCA was found to be correlated with a more malignant behavior and poorer survival outcomes in iCCA patients [31]. Consistently, other studies identified CAFs as responsible for increasing the number of tumor-initiating cells in colorectal cancer, and targeted TGF-β1, also involved in CAFs activation, reducing cancer metastasis. However, also in this case drug treatment targeted stromal cells without any evident effect on the tumor/stroma crosstalk [32]. Chimeric antigen receptors (CARs) T cells specific for FAP expressed on hCAFs have been reported to contribute to the therapeutic response in a lung tumor model, further supporting the idea of targeting CAFs because they are responsible for the deposition of the ECM components of the desmoplastic reaction [33]. We also report that Crenigacestat changed the ECM composition of the surrounding tissue microenvironment in PDX models of iCCA [22]. In accordance with our previous study, herein we also showed a reduction of the reactive stroma following Crenigacestat treatment, but in addition, we demonstrated that Crenigacestat inhibited the migration of iCCA cells only when both hCAFs and KKU-M213 and KKU-M156 were treated in co-cultured conditions simultaneously for a sufficient time to establish a cross-talk. On the contrary, Crenigacestat did not affect cell migration when the drug was used to treat hCAFs and both the iCCA cell lines each time, without being co-cultured but assembled in the assay at the end of the treatment. This phase-by-phase experimental approach further supported the hypothesis that Crenigacestat effectiveness is downstream, acting by inhibiting the tumor/stroma cross-talk. The relevance of targeting the cross-talk was also supported by the effects of Crenigacestat, arresting the cell cycle in hetero-spheroids and in the bioinformatic and transcriptomic analysis of tumor masses. This further endorses our hypothesis whereby Crenigacestat displays its effect on hCAFs/iCCA cells, leading a downstream effect on cell proliferation, downregulating cyclin E2 and arresting the cell-cycle checkpoint. This is consistent with a previous study reporting cyclin E protein overexpression in iCCA, and hence cyclin E gene as a transcriptional target of Notch signaling [34]. We hypothesize, based on our experimental data, that soluble factors released in the hCAF/iCCA cells cross-talk are likely involved in tumor progression. Therefore, the reduction of the fibrotic tissue we observed is only a part of the Crenigacestat effectiveness in targeting the hCAFs/iCCA cells cross-talk.

## Conclusions

In conclusion, we point out that the cross-talk between hCAFs and iCCA cells is a suitable target for drugs such as Crenigacestat but likely not only, thus opening out prospects for future investigations to develop new therapeutic strategies aimed at a precision medicine approach.

## Electronic supplementary material

Below is the link to the electronic supplementary material.

Supplementary Material 1–4

Supplementary Video 1

Supplementary Video 2

Supplementary Video 3

Supplementary Video 4

Supplementary Video 5

Supplementary Video 6

Supplementary Video 7

Supplementary Video 8

## Data Availability

All data generated or analysed during this study are included in this manuscript (and its supplementary information files).

## Abbreviations

Coll I: Collagen I
CK-19: Cytokeratin 19
CRE: Crenigacestat
DEG: Differentially Expressed Genes
ECM: Extracellular matrix
EpCAM: Epithelial Cell Adhesion Molecule
FAP: Fibroblast activation protein
hCAFs: human Cancer-Associated Fibroblasts
iCCA: Intrahepatic Cholangiocarcinoma
IPA: Ingenuity Pathway Analysis
PCA: Principal Component Analysis
TGF-β: Transforming Growth Factor β
TME: Tumor Microenvironment
