# Supplementary material for: Targeting cancer-associated fibroblasts/tumor cells cross-talk inhibits intrahepatic cholangiocarcinoma progression via cell-cycle arrest
Source: J Exp Clin Cancer Res. 2024 Oct 17;43:286. doi: 10.1186/s13046-024-03210-9 (PMC11484308; doi:10.1186/s13046-024-03210-9)
Supplement: Supplementary file 1 — Supplementary Material 1–4 [file 13046_2024_3210_MOESM1_ESM.docx]

**Supplementary Figure legends**


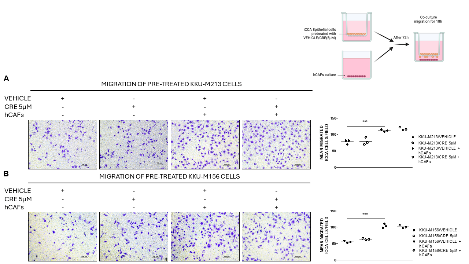


**Supplementary Figure 1. Crenigacestat did not affect the migration of pre-treated iCCA cells co-cultured with hCAFs.** Schematic representation of the transwell migration assay. iCCA cells were seeded and treated with vehicle Crenigacestat in 24-well transwell inserts of 0.4 µm pore size for 72 hours. Similarly, hCAFs were seeded in 24-wells without treatment for 72 hours. iCCA cells were subsequently transferred into 24-well transwell inserts of 8.0 µm pore size and placed in a 24-well without or with previously seeded hCAFs to form a migration chamber. Representative images of A) KKU-M213 and B) KKU-M156 cells migrated for 18 hours and then stained with crystal violet. Crenigacestat pre-treatment on KKU-M213 or KKU-M156 cells did not influence the iCCA cells migration promoted by co-culture with hCAF, compared to vehicle. The scale bar represents 100 μm. The number of migratory cells was determined in five random fields (per chamber) for each treatment. Data are expressed as the mean ± SD of three independent experiments with three hCAFs. *** p < 0.001.


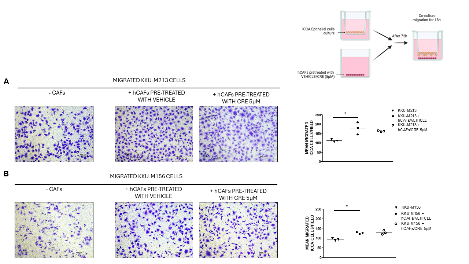


**Supplementary Figure 2. Crenigacestat did not affect the migration of iCCA cells co-cultured with pre-treated hCAFs**. Schematic representation of the transwell migration assay. hCAFs were plated and treated with vehicle or Crenigacestat in 24-wells for 72 hours. Similarly, iCCA cells were seeded in 24-well transwell inserts without treatment for 72 hours. iCCA cells were subsequently transferred into 24-well transwell inserts of 8.0 µm and seeded in 24-well inserts without or with previously seeded and treated hCAFs to form a migration chamber. Representative images of A) KKU-M213 and B) KKU-M156 cells migrated for 18 hours and then stained with crystal violet. Crenigacestat pre-treatment on hCAFs did not influence the migration of KKU-M213 or KKU-M156 cells promoted by co-culture with hCAFs, compared to vehicle. The scale bar represents 100 μm. The number of migrated cells was determined in five random fields (per chamber) for each treatment. Data are expressed as the mean ± SD of three independent experiments with three hCAFs. * p < 0.05.


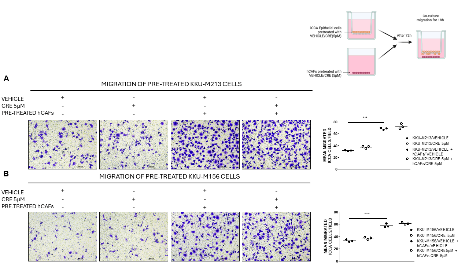


**Supplementary Figure 3. Crenigacestat did not affect the migration of pre-treated iCCA cells co-cultured with pre-treated hCAFs**. Schematic representation of the transwell migration assay. iCCA cells were seeded and treated with vehicle or Crenigacestat in 24-well transwell inserts of 0.4 µm pore size for 72 hours. Similarly, hCAFs were seeded in 24-well inserts and treated under the same conditions used for the iCCA cells, but separately from the iCCA cells. iCCA cells were subsequently transferred into 24-well transwell inserts of 8.0 µm pore size previously coated at the bottom face with Coll I and placed in a 24-well where hCAFs were previously seeded at the bottom and treated. Representative images of A) KKU-M213 and B) KKU-M156 cells migrated for 18 hours and then stained with crystal violet. Crenigacestat pre-treatment on hCAFs did not influence the migration of KKU-M213 or KKU-M156 cells promoted by co-culture with hCAFs, compared to vehicle. The scale bar represents 100 μm. The number of migratory cells was determined in five random fields (per chamber) for each treatment. Data are expressed as the mean ± SD of three independent experiments with three hCAFs. *** p < 0.001.


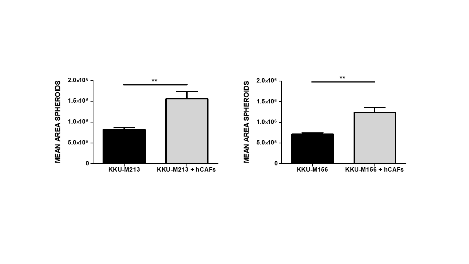


**Supplementary Figure 4. hCAFs increased the growth of 3D-model spheroids.** The spherical shape maintained after 96 hours of culture allowed the quantification of the area of 3D model spheroids by the Image J software. The analysis shows the average area of five spheroids per model, demonstrating that hCAFs increased by 2-fold the growth of heterospheroids compared to KKU-M213 or KKU-M156 spheroids.  ** p < 0.01.
